# Construction of antifungal dual-target (SE, CYP51) pharmacophore models and the discovery of novel antifungal inhibitors†

**DOI:** 10.1039/c9ra03713f

**Published:** 2019-08-22

**Authors:** Yue Dong, Min Liu, Jian Wang, Zhuang Ding, Bin Sun

**Affiliations:** Institute of BioPharmaceutical Research, Liaocheng University 1 Hunan Road Liaocheng 252000 PR China; Key Laboratory of Structure-Based Drug Design & Discovery of Ministry of Education, School of Pharmaceutical Engineering, Shenyang Pharmaceutical University 103 Wenhua Road, Shenhe District Shenyang 110016 PR China

## Abstract

Fungal infections and drug-resistance are rapidly increasing with the deterioration of the external environment. Squalene cyclooxygenase (SE) and 14α-demethylase (CYP51) are considered to be important antifungal targets, and the corresponding pharmacophore models can be used to design and guide the discovery of novel inhibitors. Therefore, the common feature pharmacophore model (SE inhibitor) and structure-based pharmacophore model (CYP51 receptor) were constructed using different methods in this study. Then, appropriate organic fragments were selected and superimposed onto the pharmacophore features, and compounds 5, 6 and 8 were designed and produced by linking these organic fragments. It is noteworthy that compound 8 can simultaneously match the features of both the SE and CYP51 pharmacophores. Further analysis found that these compounds exhibit a potent antifungal activity. Preliminary mechanistic studies revealed that compound 8 could undergo dual-target inhibition (SE and CYP51) of *Candida albicans*. This study proved the rationale of pharmacophore models (SE and CYP51), which can guide the design and discovery of new antifungal inhibitors.

## Abstract 2

Fungal infections and drug-resistance are rapidly increasing with the deterioration of the external environment.

## Graphical abstract

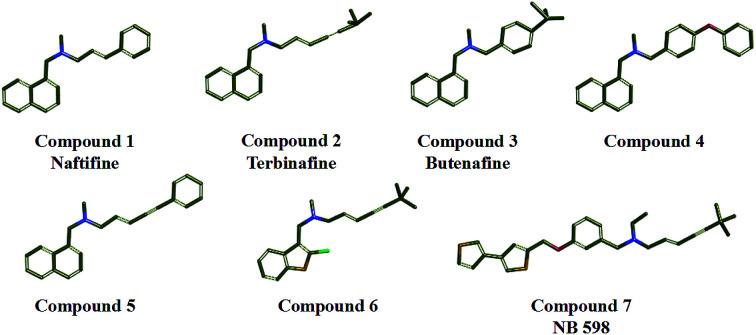

## 1. Introduction

In recent years, the number of pathogenic fungal infections and the emergence of drug-resistant fungi have increased dramatically with the deterioration of the external environment, such as the widespread abuse of broad-spectrum antibiotics, immunosuppressants, radiotherapy and chemotherapy drugs in the clinic, which results in a serious threat to human health.^1–4^*Candida* spp. is the most common conditional pathogenic fungi, which can parasitize in the human mouth, vagina and intestinal mucosa and on the skin. When the immune function of the body decreases, or the microecological environment is maladjusted in the normal place of residence, it can lead to superficial or systemic fungal infections.^5,6^ These pathogenic fungi can metabolize certain pathogenic components and toxins to cause physical injury, and reduce the ability of the body to defend itself facilitating the spread of infection.^7,8^ Ultimately, this can develop into an invasive infection, and lead to high mortality in severe cases.

Ergosterol, as an important component of the fungal cell membrane, can be blocked by inhibiting its biosynthesis.^9^ Further research revealed that rate-limiting enzymes squalene epoxidase (SE) and 14α-demethylase (CYP51) play important roles in the synthesis of ergosterol.^10–12^ If their activities are inhibited, the synthesis of ergosterol will be blocked, some upstream components and intermediates (such as 14α-methyl sterol) accumulate in the fungal cell, and eventually cause the death of the fungi.^13,14^ Currently, conventional antifungal agents can be divided into two categories based on the mode of action of the blocking ergosterol, including SE inhibitors (*e.g.*, naftifine, terbinafine and liranaftate), and CYP51 inhibitors (*e.g.*, miconazole, itraconazole and fluconazole), many of them have been developed for many years, and show good therapeutic effects in the clinic.^15–17^ However, the two kinds of inhibitors have their shortcomings, such as a narrow antimicrobial spectrum, drug resistance and low bioavailability, which negatively affect their clinical efficacy.^18–20^ Therefore, drug researchers are eager to explore the structural characteristics of SE and CYP51 inhibitors and their interaction with active sites, which could guide the discovery and design of novel antifungal inhibitors.

Pharmacophore models can summarize the structural characteristics of inhibitors based on the three-dimensional structural information of existing molecules.^21,22^ In this study, the pharmacophore models of SE and CYP51 were first constructed using different methods, respectively. Then, these pharmacophore models were summarized by analyzing their structural features, and the corresponding compounds were constructed by connecting the different organic fragments, which can be matched to the pharmacophore features. The activity and mechanisms of the target compounds are determined. This study proves the rationale for the pharmacophore model, which could play an important role in the discovery and design of new antifungal inhibitors.

## 2. Materials and methods

All computational experiments were conducted on a Dell Power Edge R910 workstation. The common feature pharmacophore model and the receptor structure-based pharmacophore (SBP) model were generated using the Pharmacophore Generation protocol in the Discovery Studio 3.0 software program (DS 3.0).^23,24^ Docking studies were performed with the CDOCKER module, and the results were shown in PyMOL.

### 2.1 Generation of the ligand-based common feature pharmacophore models

**Fig. 1:**
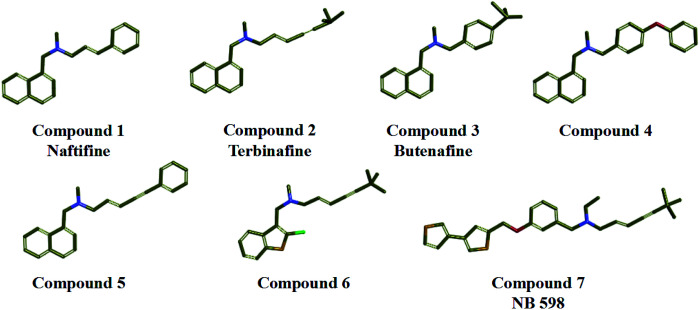
Training set compounds used in the common feature pharmacophore generation.

In the study, seven representative squalene epoxidase (SE) inhibitors with diverse scaffolds were selected to construct the training set (Fig. 1).^25–27^ For the most potent inhibitors (compounds 1, 2, 3, and 7), the principal and MaxOmitFeat values were endued with 2 and 0, respectively. For the moderate inhibitors (compounds 4, 5, and 6), the principal and MaxOmitFeat values were both set to 1. These possible pharmacophore features (H-bond acceptor (A), H-bond donor (D) and hydrophobic aliphatic (H)) were selected by analyzing the structural characteristics of the training set compounds. The value of the maximum conformation was changed to 200, the minimum features were set to 3, and the maximum features were set to 5. All other parameters were set by default. Ten pharmacophore models were produced and the best one was selected for further study.

### 2.2 Validation of the pharmacophore model

In order to evaluate the rationality and predictability of the pharmacophore model, the best pharmacophore model was selected, and it was further validated. First, the test set was constructed, which contains four randomly selected compounds with different SE inhibitory activities and three inactive compounds. Subsequently, these test set compounds were matched to the best pharmacophore model using the Ligand Profiler program in Discovery Studio 3.0. The corresponding fragments of the compounds were superimposed onto the features of the pharmacophore model, respectively. The fitting values were obtained, which can directly reflect the relationship between the matching degree and the inhibitory activity of the target compounds.

### 2.3 Generation of the CYP51 receptor structure-based pharmacophore model

**Fig. 2:**
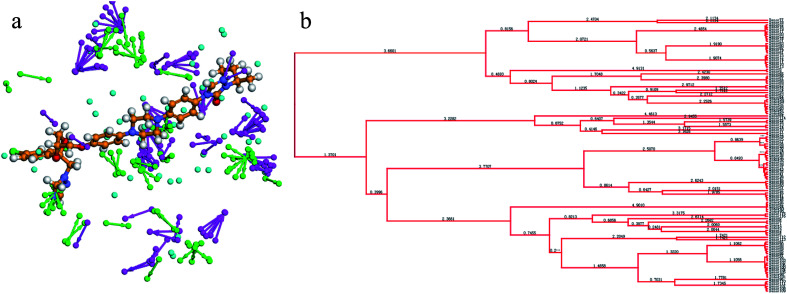
(a) The distribution of the features in the active site of CYP51. (b) The classification and clustering of the pharmacophore features.

The receptor SBP model can directly reflect the pharmacophore features of the co-crystallization ligand binding to the target enzyme. In the study, the crystal structure of CYP51 from *Candida albicans* (PDB codes: 5V5Z) was selected as the research object, and the co-crystal ligand (query on 1YN) was combined in the active cavity. First, the binding sites sphere was defined using the co-crystallized ligand 1YN, and the parameter was set to 9 Å, which comprises all of the key amino acid residues. The Interaction Generation Protocol Implemented program in DS was used to produce the HBA, HBD and HY features set based on the active site residues inside the sphere. Then, the feature interaction map was further edited, classified, clustered, and arranged. The most important feature information was retained by analyzing the ligand–receptor interaction (see Fig. 2a and b). Finally, the SBP model was obtained.

### 2.4 Synthesis of target compounds

All commercial reagents and solvents were purchased and used without additional purification. The progression of the reaction was detected using thin layer chromatography (TLC), it was performed on silica gel 60 F254 plates (Jiangyou, Yantai). Column chromatography was run on silica gel (200–300 mesh) from Qingdao Ocean Chemicals (Qingdao, Shandong, China). The melting points of all compounds were determined with a Büchi Melting Point B-540 apparatus (Büchi Labortechnik, Flawil, Switzerland) and were uncorrected. The mass spectra (MS) were determined in the electrospray ionization (ESI) mode on an Agilent 1200 LC-MS (Agilent, Palo Alto, CA, USA). Nuclear magnetic resonance (^1^H-NMR and ^13^C-NMR) spectra were recorded on a Bruker 500 MHz NMR spectrometer using tetramethylsilane (TMS) as an internal standard. The chemical shifts were reported in parts per million (ppm) and the coupling constants (*J*) were reported in Hertz (Hz). Peak multiplicities were expressed as follows: s, singlet; d, doublet; t, triplet; q, quartet; dd, doublet of doublets; dt, doublet of triplets; td, triplet of doublets; ddd, doublet of doublet of doublets; m, multiplet; and br, broad.

#### 2.4.1 General procedure for the synthesis of l-amino acid ethyl ester hydrochloride (2a–b)

l-Amino acid (alanine, serine; 1 equiv.) was dissolved in a solution of ethanol, sulfoxide chloride (3 equiv.) was slowly dropped into the mixed solution at 0 °C. Then, the mixture was heated to reflux for 3–6 h. The solvent was evaporated under reduced pressure to give a white solid.

#### 2.4.2 General procedure for the synthesis of compounds (3a and b)

The solution of 2-naphthoic acid (1 equiv.), EDCI (1.1 equiv.) and HOBt (1.1 equiv.) in DMF was slowly stirred at room temperature for 2 h. Then, l-amino acid ethyl ester hydrochloride (1.1 equiv.) and DIEA (4 equiv.) were added, and the reaction mixture was heated to 75 °C for 6 h. After confirming that the reaction was complete using TLC analysis. The reaction mixture was poured into iced water, and extracted with ethyl acetate, and the organic phase was dried over Na_2_SO_4_ overnight. Finally, the desired compound was obtained by vacuum distillation.

#### 2.4.3 General procedure for the synthesis of compound (4)

The compound (3a, 7.2 mmol) was dissolved in a 2 N sodium hydroxide (30 mL) and methanol (15 mL) solution. Then, the reaction mixture was stirred at 60 °C for 7 h. After confirming that the reaction was complete using TLC analysis, the methanol was removed by rotary evaporation. The white solids were precipitated when the pH was adjusted to 2–3 using 2 N hydrochloric acid, and it was filtered and dried to give the desired compounds.

#### 2.4.4 General procedure for the synthesis of compound (5)

To the solution of compound 3b (1 equiv.) in anhydrous CH_3_CN was added imidazole (2 equiv.) and CDI (3 equiv.). The reaction mixture was poured into water, and then extracted with EtOAc. The organic layer was dried using Na_2_SO_4_, and evaporated under reduced pressure to give the desired product. The product was purified using flash column chromatography.

#### 2.4.5 General procedure for the synthesis of compound (6)

PyBOP (1.1 equiv.) and the key intermediate (4, 1 equiv.) were added into the solution of DMF, respectively. The mixture solution was stirred at room temperature for 2 h. Subsequently, benzylamine (1.1 equiv.) and DIEA (4 equiv.) were added, the reaction mixture was heated to 80 °C and stirred for 7 h. After confirming that the reaction was complete using TLC analysis the reaction mixture was poured into iced water, the resulting solid was filtered and dried to give the desired compound. The product was purified using flash column chromatography.

#### 2.4.6 General procedure for the synthesis of compound (7)

Compound (5, 7.2 mmol) was dissolved in a 2 N sodium hydroxide (30 mL) and methanol (15 mL) solution. Then, the reaction mixture was stirred at 60 °C for 2 h. The reaction process was monitored using TLC analysis. After the completion of the reaction, the methanol was removed by rotary evaporation, pH was adjusted to 2–3 using 2 N hydrochloric acid, and the white solid was filtered and dried.

#### 2.4.7 General procedure for the synthesis of compound (8)

PyBOP (1.1 equiv.) and the key intermediate (7, 1 equiv.) were added to a solution of DMF, respectively. The reaction mixture was stirred at room temperature for 2 h. Then, benzylamine (1.1 equiv.) and DIEA (4 equiv.) were added, the reaction mixture was heated at 80 °C, and stirred for 7 h. After confirming that the reaction was complete using TLC analysis. The reaction mixture was poured into iced water, and the resulting solid was filtered and dried to give the desired compound. The product was purified using flash column chromatography.

#### 2.4.8 Ethyl (*S*)-2-(2-naphthamido)-3-(1*H*-imidazol-1-yl) propanoate (5)

Light white solid; yield: 67.2%; melting point (mp): 120.3–125.7 °C. ^1^H NMR (500 MHz, DMSO-*d*_6_) δ 9.29 (d, *J* = 7.9 Hz, 1H), 8.46 (d, *J* = 7.9 Hz, 1H), 8.06–7.99 (m, 3H), 7.94 (d, *J* = 1.1 Hz, 1H), 7.76 (s, 2H), 7.63 (d, *J* = 1.5 Hz, 1H), 7.29 (s, 1H), 6.92 (s, 1H), 4.94 (td, *J* = 9.4, 5.1 Hz, 1H), 4.56 (ddd, *J* = 23.8, 14.0, 7.4 Hz, 2H), 4.18 (q, *J* = 7.1 Hz, 2H), 1.21 (t, *J* = 7.1 Hz, 3H). ^13^C NMR (126 MHz, DMSO) *δ* 167.19, 138.36, 135.65, 134.79, 132.52, 131.27, 129.35, 128.77, 128.51, 128.32, 128.28, 128.12, 127.35, 124.53, 122.11, 120.45, 61.58, 54.37, 46.38, 14.44. ESI-MS *m*/*z*: 338.2 [M + H]^+^; 360.3 [M + Na]^+^; 337.1 [M − H]^−^. HRMS calculated for C_19_H_17_N_3_O_2_, [M + H]^+^, 338.14264; found 338.14529.

#### 2.4.9 (*S*)-*N*-(1-(Benzylamino)-1-oxopropan-2-yl)-2-naphthamide (6)

Light white solid; yield: 70.3%; mp: 135.9–146.2 °C. ^1^H NMR (500 MHz, DMSO-*d*_6_) δ 8.75 (d, *J* = 7.3 Hz, 1H), 8.55 (dd, *J* = 15.6, 9.8 Hz, 2H), 8.06–7.98 (m, 4H), 7.64–7.58 (m, 2H), 7.38–7.23 (m, 5H), 4.62 (td, *J* = 7.2 Hz, 1H), 4.35 (ddd, *J* = 6.0 Hz, 2H), 1.45 (d, *J* = 7.2 Hz, 3H). ^13^C NMR (126 MHz, DMSO-*d*_6_) δ 173.00, 166.69, 140.03, 134.66, 132.58, 131.94, 129.32, 128.78, 128.71, 128.32, 128.17, 128.09, 127.78, 127.47, 127.18, 127.14, 125.00, 49.77, 42.50, 18.55. ESI-MS *m*/*z*: 333.2 [M + H]^+^; 355.3 [M + Na]^+^; 331.2 [M − H]^−^. HRMS calculated for C_20_H_19_N_3_O_2_, [M + H]^+^, 333.15248; found 333.15104.

#### 2.4.10 (*S*)-*N*-(1-(Benzylamino)-3-(1*H*-imidazol-1-yl)-1-oxopropan-2-yl)-2-naphthamide (8)

Light white solid; yield: 71.8%; mp: 139.3–146.3 °C. ^1^H NMR (500 MHz, DMSO-*d*_6_) *δ* 8.97 (d, *J* = 8.5 Hz, 1H), 8.76 (t, *J* = 5.9 Hz, 1H), 8.46 (s, 1H), 8.10–7.79 (m, 4H), 7.79–7.41 (m, 3H), 7.28 (ddd, *J* = 26.0, 16.3, 9.0 Hz, 6H), 6.83 (s, 1H), 4.97 (td, *J* = 9.6, 4.4 Hz, 1H), 4.56–4.37 (ddd, 2H), 4.34 (d, *J* = 14.5, 8.1 Hz, 2H). ^13^C NMR (126 MHz, DMSO-*d*_6_) *δ* 169.56, 167.03, 139.51, 138.22, 134.72, 132.49, 131.56, 129.34, 128.76, 128.31, 128.23, 128.11, 127.65, 127.30, 124.75, 120.24, 55.18, 49.07, 47.37, 42.75. ESI-MS *m*/*z*: 399.2 [M + H]^+^; 421.1 [M + Na]^+^; 397.2 [M − H]^−^. HRMS calculated for C_22_H_23_N_3_O_2_, [M + H]^+^, 399.17428; found 399.17076.

### 2.5 Molecular docking

Molecular docking can assist the evaluation of the binding mode of inhibitors and receptors, and guide the optimization of lead compounds. In this study, the three-dimensional structure of SE was constructed using the homology modeling method, and the crystal structure of CYP51 has been previously reported. They were selected as the receptors, respectively. First, these receptor proteins (SE, CYP51) were endowed with the hydrogen atoms and the force field of CHARMm. The position of the co-crystalline ligand is defined as the active site, and the parameter of the active sphere was set to 9 Å. At the same time, the target compounds were also optimized to obtain conformations with the lowest energy. Then, the target compounds were docked into the active site using the CDOCKER program. The maximum save conformation was set to 10, and all other options were kept as the default settings during the docking process. Finally, the docking result was obtained.

### 2.6 *In vitro* antifungal activity test

The antifungal activity of the target compounds was determined using the standard guidelines, which are described in the National Committee for Clinical Laboratory Standards (NCCLS), and the *in vitro* minimum inhibitory concentration (MIC) value is defined as the lowest concentration of an antifungal inhibitor with an inhibitory effect. Fluconazole and terbinafine were selected as positive control drugs. In the study, the pathogenic fungi were cultured in Sabouraud medium, and all of the target compounds were dissolved in DMSO, they were serially diluted into the medium, and this concentration was configured with a fixed gradient. The daily growth of the fungi was monitored under 35 °C culture conditions.

### 2.7 Analysis of the components of *C. albicans* cells

In this process, *Candida albicans* (ATCC SC5314) was selected as the test strain. Fluconazole and terbinafine were purchased as the positive control drugs. In the study, the concentration of the drug was set to 8 μg mL^−1^. After 32 h of cultivation, the different groups of wet bacteria were concentrated and washed with PBS, and the sterol component was extracted using petroleum ether. The solvent was removed under reduced pressure. Finally, the product was dissolved in methanol solution (10 mL), filtered and detected using high performance liquid chromatography (HPLC). The chromatographic conditions were selected with a Phenomenex Luna C_18_ (2.50 × 4.6 mm, 5 μm) column. The mobile phase was methanol : water (98 : 2), the flow rate was 1 mL min^−1^, and the detection wavelength was 210 nm. The peak area ratio of each component was calculated.

### 2.8 ADMET prediction

The properties of ADMET include the absorption, distribution, metabolism, excretion and toxicity of drugs in the human body. ADMET prediction can guide the selection and optimization of leading compounds in the early stages of drug development, the specific operation process is as follows: select the “ADMET descriptors” module, and open the parameter browser. The small molecule compound files are imported. In the parameter settings, the aqueous solubility, blood brain barrier penetration, CYP2D6 binding, hepatotoxicity, intestinal absorption and plasma protein binding were chosen as research objects, respectively.

## 3. Results and discussion

### 3.1 Common feature pharmacophore modeling (Hip-Hop) of SE inhibitors and validation

A common feature pharmacophore model, which is known as an effective tool to identify and discover novel compounds with good biological activity, can be constructed *via* the Hip-Hop program in Discovery Studio software. The seven representative SE inhibitors with different scaffolds were selected as the training set. Finally, all of the top ten pharmacophore hypotheses models were produced, and they all contain three hydrophobic groups (H), which indicate that these SE inhibitors exhibit similar hydrophobic properties. The statistical parameters are listed in Table 1, and the range of the rank scores was from 75.422 to 78.922 kcal mol^−1^. Meanwhile, the maximum matching value of max fit is 4, the direct hit (1111111) and partial hit (0000000) values show that all of the training set compounds can match the chemical features of pharmacophore model. The result suggested that all 10 hypotheses models have a similar style of spatial arrangement.

**Table 1:** Summary of the pharmacophore models generated by Hip-Hop for SE inhibitors

Hypothesis	Features^a^	Rank^b^	Direct hit^c^	Partial hit^d^	Max fit
01	HHH	78.922	1111111	0000000	3
02	HHH	78.233	1111111	0000000	3
03	HHH	78.233	1111111	0000000	3
04	HHH	77.988	1111111	0000000	3
05	HHH	77.959	1111111	0000000	3
06	HHH	77.344	1111111	0000000	3
07	HHH	76.844	1111111	0000000	3
08	HHH	76.692	1111111	0000000	3
09	HHH	75.466	1111111	0000000	3
10	HHH	75.422	1111111	0000000	3

a H, hydrophobic group.

b Ranking score of the training set compounds fitting the hypothesis.

c Direct hit indicates whether (“1”) or not (“0”) a molecule in the training set mapped every feature in the hypothesis.

d Partial hit indicates whether (“1”) or not (“0”) a particular molecule in the training set mapped all but one feature in the hypothesis. Numeration of molecules is from right to left in both direct hit and partial hit.

Then, the constructed hypotheses (hypo) models were distinguished by the rank scores and the fit values of the training set compounds. The hypo-01, -02, -04, and -06 exhibited better matching properties than hypo-03, -05, -07, -08, -09, and -10. In order to determine the optimal pharmacophore model, the hypo-01, -02, -04, and -06 were further investigated, and the training set compounds were able to match the hypo 01, 02, 04, and 06, respectively. The corresponding fit values of the training set compounds are shown in Table 2, the hypo 02 model was indicated as the best and final ligand-based pharmacophore model, which can predict the activity of different compounds by evaluating the changes in the fitting values.

**Table 2:** Validation of hypotheses 01, 02, 04 and 06 by scaling the fit values, and the conformation values of the compounds in the training set^a^

Training set compound	Bioactivity values	Scaled-fit values^b^			
	IC_50_ [nm]	Hypo 01	Hypo 02	Hypo 04	Hypo 06
1	13	3.468	3.243	3.149	3.377
2	24	2.993	3.037	2.875	2.965
3	18	3.106	3.037	2.978	2.976
4	150	2.916	2.822	2.768	2.765
5	247	2.785	2.917	2.653	2.726
6	258	2.859	2.738	2.746	2.796
7	35	2.879	2.972	2.968	2.843

a Diverse set of conformations for all molecules was generated by using ‘Best conformer generation’ option with 20 kcal mol^−1^ energy cutoffs.

b Compare fit analysis of all compounds with maximum omitting feature value as 1.

**Fig. 3:**
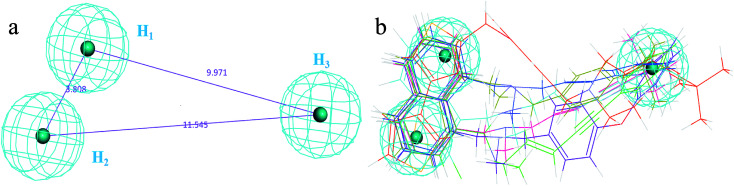
(a) Selected common-feature pharmacophore model hypothesis 02 consisting of three chemical features (HHH). Distances between the features are expressed in Å, with a tolerance sphere of radius ± 0.9 Å. (b) The majority of training set compounds were matched to the chemical features of hypo-02.

The 3D spatial relationship and geometric parameters of hypo-02 are depicted in Fig. 3a and b, which contains three hydrophobic features (H_1_, H_2_, H_3_). Clearly, the training set compounds can be matched to the pharmacophore features, and these features of the model are located in the substituted phenyl groups and the hydrophobic groups. The potent inhibitors such as compounds 1, 2 and 3, but not compound 7, present a good matching effect. For the moderately active compounds 4, 5 and 6, their structural fragments could not match all of the pharmacophore features at the same time.

In order to further verify the reliability and predictability of hypo-02, the training set was constructed using the randomly selected four potent SE inhibitors (1–4) and three inactive compounds (5–7), they were matched to hypo-02 using the program of ligand pharmacophore mapping, and the results are shown in Table 3. The range of corresponding align/fit values is from 0.967 to 3.154. The inactive compounds with low fit values were unable to match the two-hydrophobic (H) features at the same time, and the fit values of the potent SE inhibitor (1–4) are significantly higher than for the inactive compounds. From the results described above, it is revealed that hypo-02 is a reliable and predictable pharmacophore model, and it can be applied to discover and identify novel antifungal inhibitors.

**Table 3:** Mapping of the test set compounds onto hypothesis 02

Test set compound	3D mapping onto hypo-02	*E* (kcal mol^−1^)	Fit value	MIC, μg mL^−1^
1	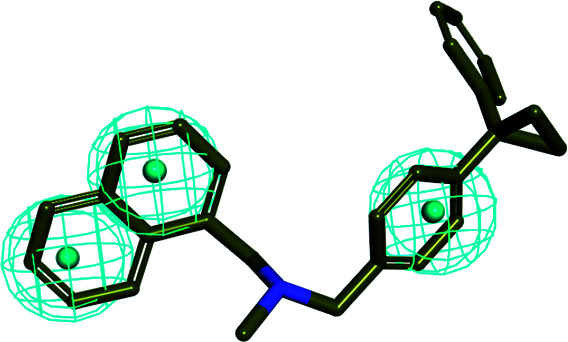	41.432	3.154	3 (ref. 28)
2	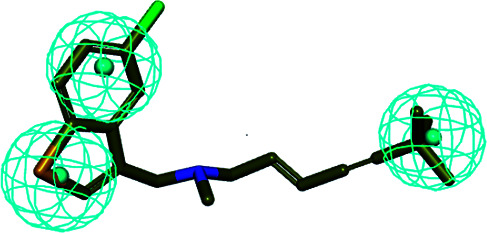	36.542	2.945	50 (ref. 29)
3	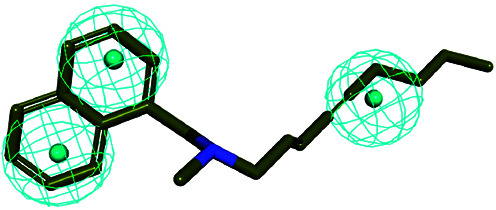	43.639	2.793	100 (ref. 25)
4	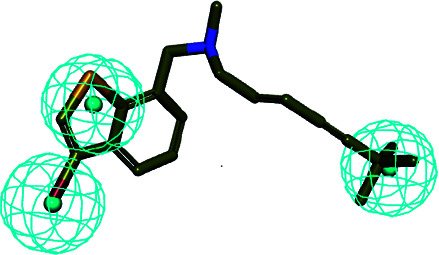	34.424	2.980	800 (ref. 29)
5	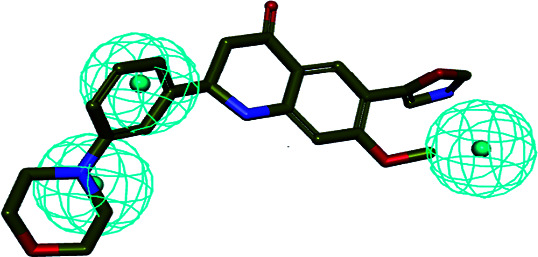	37.562	2.031	—^30^
6	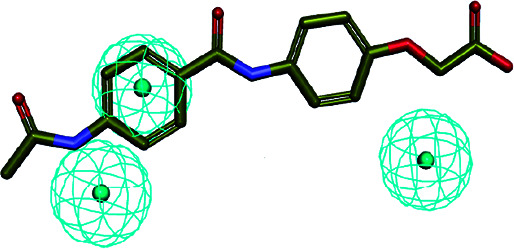	26.879	1.057	—^30^
7	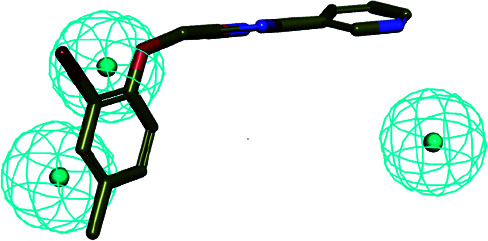	25.104	0.967	—^30^

### 3.2 Selection of the crystal structures of CYP51 and analysis of common sets of water molecules

**Fig. 4:**
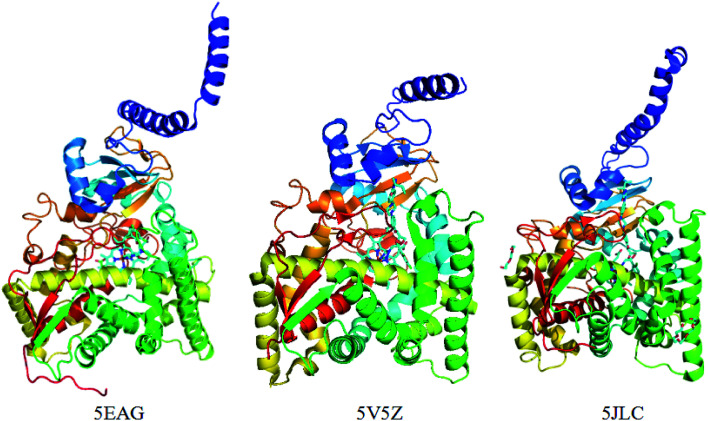
The crystal structure of the CYP51 protein with different co-crystallizing ligands.

Previously, the crystal structure of CYP51 with different co-crystallizing ligands from the pathogen *Candida albicans* has been reported (PDB code: 5TZ1 (ref. 31*a*), 5V5Z,^31*b*^5JLC^31*b*^) (Fig. 4). The X-ray resolutions of the three crystalline proteins are 2.0, 2.9 and 2.4 Å, respectively. In order to select the suitable crystal structure of CYP51, docking verification was performed in the presence and absence of water molecules. This result shows that molecular docking is similar in the presence and absence of water molecules. The optimal crystal protein with the highest score (90.67) and the lowest root mean square deviation (RMSD) value (0.45) is 5V5Z in the absence of water molecules, which demonstrates external water molecules cannot influence the conformation of the binding ligand (Table 4). At the same time, the ligands can form a π-alkylation interaction with key amino acid residues in the crystal structures, the result initially indicates the hydrophobic properties of theCYP51 active cavity. Therefore, the crystal structures of CYP51 (5V5Z) with no water shows excellent characteristics and can be selected for in-depth study.

**Table 4:** Summary of native docking

PDB Code	Resolution [Å]	Water set			No water set		
		Score	RMSD	Key residues^a^	Score	RMSD	Key residues
5TZ1	2.0	78.764	0.53	Tyr126, Gly314, Leu380	80.36	0.47	Tyr126, Gly314, Leu380
5V5Z	2.9	86.752	0.45	Ile131, Pro230, Leu376, His377	90.67	0.41	Ile131, Pro230, Leu376, His377
5JLC	2.4	69.273	0.74	Pro239, Phe242, Leu381, His382	72.46	0.68	Pro239, Phe242, Leu381, His382

a Residues interacting with the docked poses through hydrogen bonds.

### 3.3 Analyzing the receptor structure-based pharmacophore model of CYP51

The SBP model can directly obtain the feature interaction map of the ligand from the receptor sites, and the information from the site map can be transformed into the three-dimensional pharmacophore model, which can directly reflect the reasonable interaction between the ligand and protein.

**Fig. 5:**
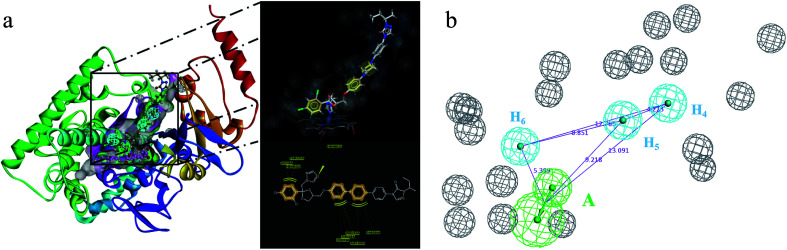
(a) Constructed structure-based pharmacophore model consisting of ten chemical features; and (b) the distribution of the pharmacophore model features in the active sites.

In the cluster process of the pharmacophore features, the hydrophobic features occupy the main region of the co-crystalline ligand, and hydrogen bond acceptor features are mainly located in the imidazole group region, in which the hydrogen-bonded donor features are far away from the co-crystalline ligand molecules. Therefore, the hydrogen bond acceptor and hydrophobic features are retained, and the hydrogen-bonded donor features are abandoned, which is depicted in Fig. 5a and b. Its structure contains four chemical features: three hydrophobic (H) and one hydrogen bond acceptors (A) features, which are distributed in the different structural fragments of the co-crystallized ligand (itraconazole). The main region of the ligand contains aromatic fragments, which can form hydrophobic interactions with the key hydrophobic amino acids in the CYP51 active cavity. Therefore, the hydrophobic (H_4_, H_5_, and H_6_) features are produced, and are located in this region. In addition, it can be obviously observed that the imidazole group of the ligand can form an important coordination bond interaction with the heme at the bottom of the active chamber. Accordingly, the hydrogen bond acceptors (A) feature was produced, which can be matched to the imidazole group.

### 3.4 Design and synthesis of antifungal agents

**Fig. 6:**
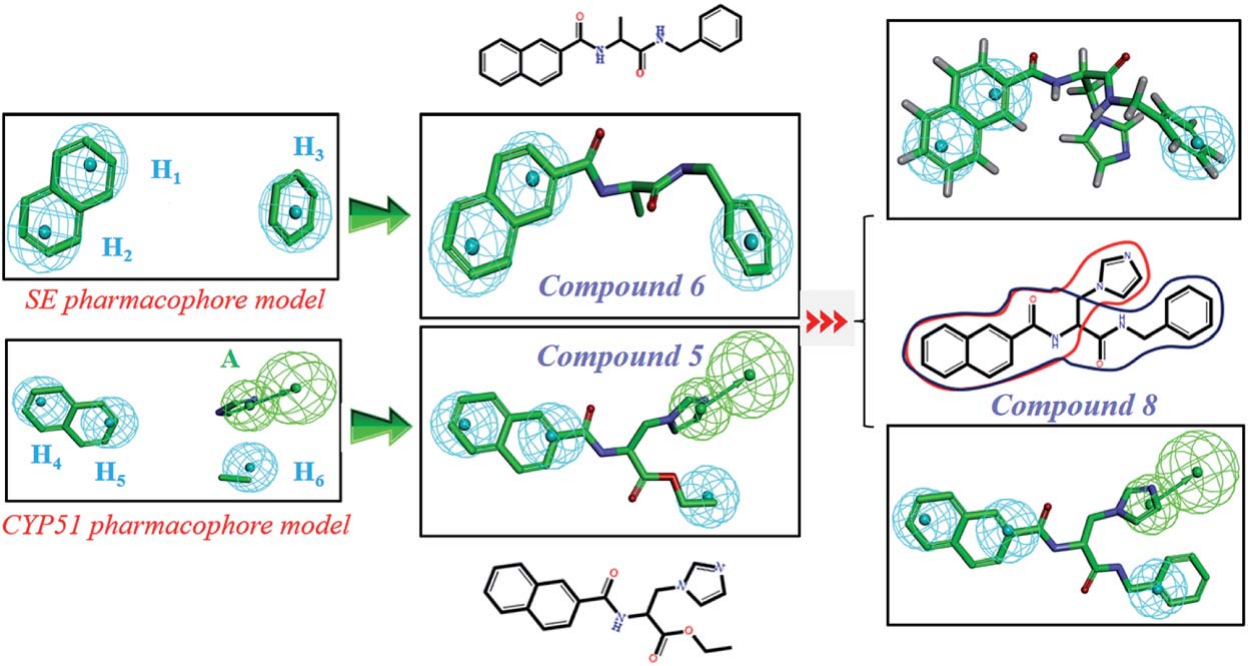
Design of novel SE inhibitors (compound 6), CYP51 inhibitors (compound 5) and dual-target inhibitors (compound 8) based on the pharmacophore features.

In order to discover the novel antifungal inhibitors based on the constructed SE and CYP51 pharmacophore models, the strategy was to match the different pharmacophore features with specific organic fragments (Fig. 6). In the SE pharmacophore model, the naphthyl fragment was retained, which can be matched to the hydrophobic groups H_1_ and H_2_. In addition, the phenyl fragment was positioned at the hydrophobic group H_3_. Subsequently, the target compound 6 was constructed by connecting the naphthyl fragment and the phenyl fragment, and it was expected to have a SE inhibitory activity. In the CYP51 pharmacophore model, the naphthyl fragment could also be simultaneously matched to the hydrophobic groups H_4_ and H_5_. The ester fragment was positioned at the hydrophobic group H_6_, and the relatively small imidazole fragment was located at the hydrogen bond acceptors (A) feature, so that the target compound could form the essential coordinate bond with the ferrous ion of the heme group. Subsequently, the novel target compound 5 was constructed by connecting each part of the fragment structures, and it was expected to have a CYP51 inhibitory activity. Next, the SE and CYP51 pharmacophore models were further analyzed, their structural characteristics are similar in terms of their spatial distribution. Therefore, the naphthyl fragments of compounds 6 and 5 were retained in the same region, and the specific phenyl fragment (SE) and imidazole fragment (CYP51) were connected by serine. Finally, the target compound 8 was constructed, it was expected that the compound may have a simultaneous double-target (SE, CYP51) inhibitory activity, and the following experimental data confirmed the hypothesis of our design.

**Scheme 1:**
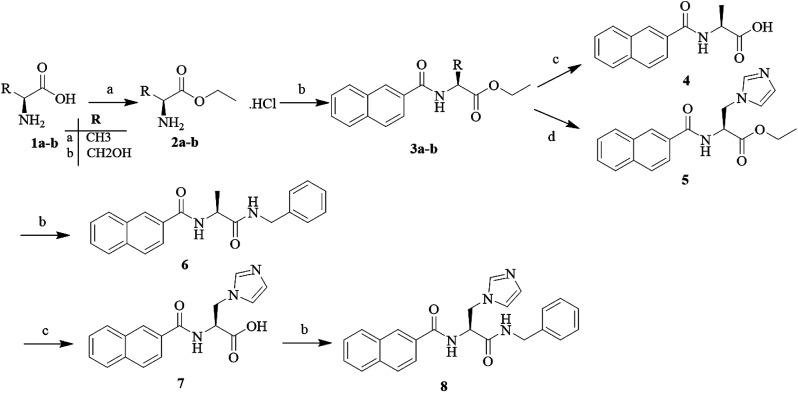
Reagents and conditions: (a) alcohol reagent, SOCl2, reflux, 2–3 h; (b) EDCI, HOBt, DIEA, 2-naphthoic acid, 75 °C, 7 h; (c) 2 N NaOH solution, MeOH, 60 °C, 5 h; and (d) CDI, imidazole, DMF, reflux, 7 h.

The target compounds 5, 6 and 8 were prepared according to the synthetic route outlined in Scheme 1. First, l-alanine and l-serine (1a and b) were dissolved in ethanol and refluxed with SOCl_2_ for 3 h, and the amino acid ethyl ester hydrochlorides (2a and b) were obtained, respectively. Next, these amino acid ethyl ester hydrochlorides were separately treated with the key intermediate 2-naphthoic acid in the presence of a condensing agent to give the required products (3a and b). Subsequently, the compound 3a was hydrolyzed with 2 N sodium hydroxide solution to obtain the corresponding organic acids (4), and the compound 3b was treated to obtain the target compound (5) with CDI and imidazole. Part of the target compound 5 was further hydrolyzed to obtain the intermediate compound 7. Finally, the target compound 8 was synthesized from the organic acid and benzylamine *via* an amidation reaction.

### 3.5 Molecular docking

In order to further validate the binding modes between the target compounds (5, 6 and 8) and the target enzymes (SE and CYP51), molecular docking was performed using the CDOCKER program in Discovery Studio 3.0. The 2D images depicting the proposed binding modes were produced using LigPlus.

The docking results are shown in Table 5. The range of CDOCKER energy values and interaction energy were predicted as being −25.4 to −38.56 kcal mol^−1^ and −33.24 to −43.23 kcal mol^−1^, respectively. The binding energies of the target compounds were close to or lower than these reference molecules (terbinafine and fluconazole), which suggests that these compounds can properly combine with their respective active cavities. Moreover, the ranges of the RMSD are 0.72–1.12 Å by comparison with the binding conformation of the reference molecules, which indicated the compounds have a similar binding mode to the reference molecules.

**Table 5:** Summary of the docking studies of the target compounds

Compound	Target enzyme	-CDOCKER energy	-CDOCKER interaction energy	Coordinate bond-distance (Å)	RMSD (Å)
5	CYP51	34.02	43.23	2.71	0.72
6	SE	25.4	37.17	—	0.95
8	CYP51, SE	31.27, 27.6	35.65, 32.5	2.82	1.12, 1.05
Terbinafine	SE	28.71	33.24	—	—
Fluconazole	CYP51	38.56	41.56	2.34	—

**Fig. 7:**
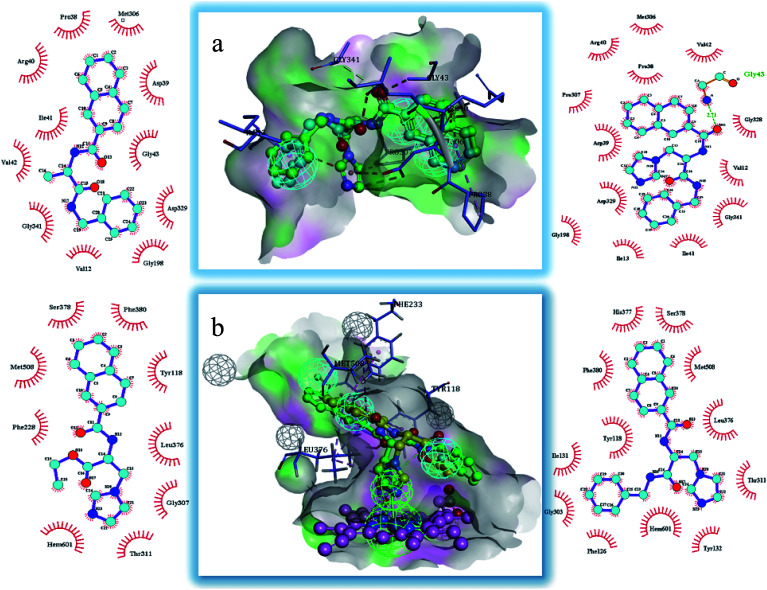
Docking models of the representative compounds. (a) The binding mode of compounds 6 and 8 in the active site of SE. (b) The binding mode of compounds 5 and 8 in the active site of CYP51.

The binding modes of the target compounds were further studied (Fig. 7), target compounds 6 and 8 were docked in the SE enzyme, the various fragments of the compounds can properly match to the active chamber of SE, their phenyl fragments can form a π-alkylation with Ile13, and the naphthyl fragments form π–π or a π-alkylation with Pro38, Arg40 and Met306. At the same time, the flexible branched chain of compound 8 containing an amide bond group can produce a hydrogen bonding interaction with Gly43. In addition, the target compounds 5 and 8 can also be docked into the active cavity of CYP51. The imidazole fragments of the compounds occupy the bottom region of the active cavity, and the coordinate bond interactions were formed with the heme group of CYP51 (2.71 Å, 2.82 Å), which plays an important role in achieving the high affinity. The naphthyl fragments occupy the upper region of the active cavity, and they can produce the hydrophobic interactions (π–π or π-alkylation) with the key residues Pro 230, Phe 233, Leu376 and Met508. The ester fragment of compound 5 and the benzyl fragment of compound 8 are bound to the lower region of the active cavity, which can enhance the inhibitory activity of the compounds by producing interactions with the surrounding key amino acid residues.

### 3.6 *In vitro* antifungal activity

The *in vitro* antifungal activity of the target compounds was detected according to the protocols from the NCCLS, the pathogenic *Candida* (*C. albicans*, *C. glabrata*, *C. krusei*, and *C. tropicalis*), Aspergillus fungi (*A. fumigatus*) and fluconazole-resistant strains of *C. alb.* (strain 100 and strain 103) were selected as test strains, respectively. The minimum inhibitory concentrations (MICs) of the target compounds in 96-well micro test plates can reflect the inhibition ability of the target compounds.

The results are listed in Table 6. It can be seen that all of the target compounds exhibited moderate to good inhibitory activities against *Candida* and *Aspergillus fumigatus*, which suggests the rationality of the pharmacophore model (SE, CYP51). The inhibitory activities of compounds 5 and 6 can be observed simultaneously on *C. gla.*, *C. kru.* and *C. tro.* (MIC range: 0.5–2 μg mL^−1^), which is similar to fluconazole and terbinafine, however, their inhibitory activity on *Aspergillus fumigatus* show a significant decrease. In addition, the target compound 8 also showed broad spectrum antifungal activity, and its inhibitory activity on partial *Candida* such as *C. alb. C. gla.* was better than that of fluconazole and terbinafine. It is noteworthy that compound 8 can also significantly inhibit *Aspergillus fumigatus* (MIC range: 2 μg mL^−1^). At present, the problem of fungal drug resistance is becoming more and more serious. The target compounds 5, 6 and 8 were further evaluated against fluconazole-resistant strains of *C. alb.* (strain 100 and strain 103). They showed moderate antifungal activities against strain 100 and strain 103, with MIC values in the range of 1–4 μg mL^−1^.

**Table 6:** *In vitro* antifungal activities of the target compounds (MIC, μg mL^−1^)^a^

Compound	MIC, μg mL^−1^						
	*C. alb.*	*C. gla.*	*C. kru.*	*C. tro.*	*A. fum.*	*C.alb.* strain 100	*C.alb.* strain 103
5	0.5	0.25	1	0.5	>16	4	4
6	1	1	0.5	2	8	4	2
8	0.25	0.5	1	0.5	2	2	1
Fluconazole	0.5	1	0.5	2	>16	>16	>16
Terbinafine	0.25	1	0.5	2	4	4	8

a Abbreviations: *C. alb.*, *Candida albicans* (ATCC 10231); *C. gla.*, *Candida glabrata* (ATCC 0001); *C. kru*., *Candida krusei* (ATCC 6258); *C. tro.*, *Candida tropicalis* (ATCC 1369); *A. fum.*, *Aspergillus fumigatus* (KM8001). Strain 100, fluconazole-resistant strains of *Candida albicans*; strain 103, fluconazole-resistant strains of *Candida albicans*; strain 100 and strain 103 were provided by the Second Military Medical University.

### 3.7 Morphological analysis of *C. alb.* strains with different target compounds

**Fig. 8:**
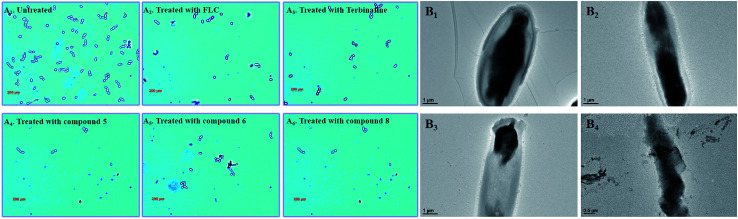
(A_1–6_) Polarizing microscopy results of *Candida albicans* treated with the positive control drugs (fluconazole and terbinafine) and the target compounds (5, 6 and 8) at the specific concentration of 8 μg mL. (B_1–4_) TEM results for *Candida albicans* treated with the target compound (8) at the specific concentration of 8 μg mL^−1^.

In the untreated group, the cell density of *Candida albicans* increased obviously (Fig. 8A), with some fungal cells forming spores in the division stage. In the treatment groups, the target compounds (5, 6 and 8) show similar pharmacodynamic effects as the positive control drugs (fluconazole and terbinafine) at a concentration of 4 μg mL^−1^, which can inhibit the proliferation of *Candida*. They can not only effectively reduce cell division, but also kill existing *Candida* cells. In particularly, for the treatment group for compound 8, the density of *Candida albicans* was even lower than the treatment groups for the positive control drugs. In order to further understand the effect of compound 8 on the cell morphology, the morphological changes of the *Candida* cells were observed using TEM at different time intervals (Fig. 8B). At the initial stage, the cell morphology of *Candida albicans* was elliptical, the cell wall and cell membrane were clearly and transparently covered on the cell surface, and the internal cytoplasm was evenly distributed. When the fungal cells were treated with compound 8 for 4 d, the cell morphology showed columnar changes. The cell wall and the cell membrane began to disappear, and the internal cytoplasmic distribution became uneven. The phenomenon may be caused by blocking of the synthesis of ergosterol. This eventually destroyed the cell permeability, and external substances began to infiltrate into the cell interior. Then, the fungal cells began to rupture, and the internal material seeped out of the cells. Ultimately, only residues of the fungal cell wall and cell membrane were left.

### 3.8 Analysis of antifungal mechanisms

In the synthetic pathway of ergosterol, inhibition of the SE and CYP51 activity can cause the aggregation of the upstream composition. Therefore, HPLC was used to detect changes to the corresponding composition of *Candida albicans*, and fluconazole and terbinafine were selected as the reference drugs.

The results are shown in Table 7. In the untreated group, ergosterol was measured as the main peak, which accounted for 99.38% of the extract product, and squalene comprised only 0.62% of the total contents. In the fluconazole and compound 5 treatment groups, the lanosterol and squalene increased significantly, and the ergosterol content decreased to 2.57% and 0.81%, respectively. The result suggests that compound 5 exhibits the same action mechanism as fluconazole by inhibiting CYP51. In the terbinafine and compound 6 treatment groups, the squalene content rose to 67.07% and 64.75%, respectively. The ergosterol content reduced to 6.54% and 15.09%, which indicates that compound 6 exhibits an antifungal activity mainly by inhibiting SE. In the compound 8 treatment group, it is worth noting that the ergosterol content decreased to 9.02%, while the lanosterol and squalene contents increased to 30.57% and 54.84% of the total product, respectively. The result gives preliminarily proof that compound 8 can exhibit a moderate dual-target (SE, CYP51) inhibition activity.

**Table 7:** Analysis of composition in *C. albicans*

Contents	Percentage of total sterols (*Candida albicans*)					
	Control	Fluconazole	Terbinafine	5	6	8
Ergosterol	99.38	2.57	6.54	0.81	15.09	7.79
Lanosterol	—	75.96	—	78.09	—	30.57
Squalene	0.62	20.18	67.07	5.11	64.75	54.84
Unidentified	—	1.3	26.39	15.98	20.16	6.8

### 3.9 Theoretical evaluation of ADMET properties

The proper ADMET properties can greatly increase the success rate of new drug development, and reduce the wasting of funds. Therefore, the ADMET values of the target compounds were predicted and analyzed.

**Fig. 9:**
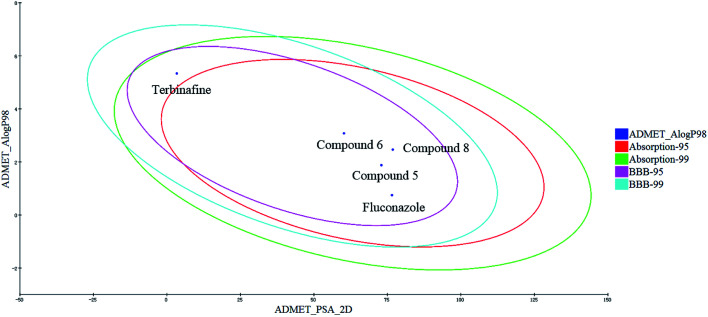
Plot of the PSA *versus* Alog *P* (the logarithm of the partition coefficient between octanol and water) for the candidate compounds.

The human intestinal absorption (HIA) and blood–brain barrier (BBB) penetration models are expressed by the two analogous 95% and 99% confidence ellipses (Fig. 9). All of the target compounds and the control drug (fluconazole) were distributed in the 99% confidence region for the BBB penetration and HIA, and terbinafine was excluded from the 95% confidence ellipses of HIA (absorption-95), which suggests that some properties of the target compounds are even better than those for terbinafine.

The detailed results are shown in Table 8. When the value is less than 5, it indicates that the ADMET for the compounds is located at a reasonable interval. The range of the BBB for the compounds is −1.155 to −0.607, which indicate these compounds have a low BBB penetration; and they can increase the drug action time by inhibiting CYP2D6, compounds 5 and 6 have an even smaller hepatotoxicity than fluconazole and terbinafine. In addition, the range of the solubility for the compounds (−3.673 to −3.21) and the Alog *P* (1.629 to 3.079) are also located in the allowed range, which can directly reflect the rational absorption and permeation of compounds. Likewise, the polar surface area (PSA) parameter can directly reflect the drug bioavailability. When the PSA values of the compounds are all less than 140, these compounds can retain reasonable properties for bioavailability. Therefore, these compounds can be used as a starting point for further developing and designing new derivatives.

**Table 8:** *In silico* ADMET prediction of the potential compounds 5, 6 and 8 compared with terbinafine and fluconazole

ADMET parameters	5	6	8	Fluconazole	Terbinafine
ADMET-BBB	−0.728	−0.155	−0.607	−1.134	1.442
ADMET-solubility	−3.21	−3.496	−3.673	−1.976	−6.125
ADMET-hepatotoxicity	−0.559	−4.710	0.286	11.222	−0.378
ADMET-CYP2D6	−3.23	−3.23	−1.933	−13.977	17.1148
Alog *P*98	1.879	3.079	1.629	0.750	5.335
PSA	72.951	60.222	76.831	76.556	3.352

## 4. Conclusions

With the deterioration of the external environment, fungal infections are becoming more and more frequent in clinical practice. The rapid discovery of new antifungal drugs has gradually become an urgent problem that needs to be solved. The corresponding pharmacophore models can be used to design and guide the discovery of novel inhibitors. Firstly, the common feature pharmacophore (Hip-Hop) model of the SE inhibitors was constructed using eight representative SE inhibitors as the training set, all of the top ten pharmacophore hypotheses models were produced, the hypo-02 contains three hydrophobic features (H), and it was selected and validated as the final pharmacophore model, and can be used to distinguish between the active and inactive compounds. On the other hand, the optimal protein crystal 5V5Z was selected to produce the SBP model by analyzing the binding conformation between the ligand and CYP51 active cavity, the CYP51 pharmacophore model was constructed, and it contains four chemical features: three hydrophobic (H) and one hydrogen bond acceptor (A). Subsequently, different active fragments were matched to the two pharmacophore features, the novel antifungal compounds (5, 6 and 8) were designed and produced using the method of fragment-based drug discovery. These target compounds show an excellent antifungal activity, in particular compound 8 can cause the splitting decomposition of fungal cells. The preliminary action mechanism proves that these target compounds can affect the activity of the corresponding target enzymes. It is worth noting that compound 8 can block ergosterol synthesis by dual-target (SE and CYP51) inhibition. This study proves the rationale of the pharmacophore model, which can play an important role in the design and discovery of novel antifungal inhibitors.

## Conflicts of interest

There are no conflicts to declare.

## Supplementary Material

RA-009-C9RA03713F-s001
