# Correction: Construction of antifungal dual-target (SE, CYP51) pharmacophore models and the discovery of novel antifungal inhibitors

**DOI:** 10.1039/d4ra90079k

**Published:** 2024-07-26

**Authors:** Yue Dong, Min Liu, Jian Wang, Zhuang Ding, Bin Sun

**Affiliations:** a Institute of BioPharmaceutical Research, Liaocheng University 1 Hunan Road Liaocheng 252000 PR China; b Key Laboratory of Structure-Based Drug Design & Discovery of Ministry of Education, School of Pharmaceutical Engineering, Shenyang Pharmaceutical University 103 Wenhua Road, Shenhe District Shenyang 110016 PR China

## Abstract

Correction for ‘Construction of antifungal dual-target (SE, CYP51) pharmacophore models and the discovery of novel antifungal inhibitors’ by Yue Dong *et al.*, *RSC Adv.*, 2019, **9**, 26302–26314, https://doi.org/10.1039/c9ra03713f.

The authors regret that an incorrect version of Fig. 8 was included in the original article. The correct version of Fig. 8 is presented here.
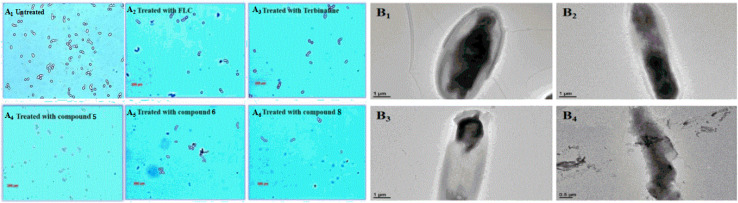


**Fig. 8** (A_1_–_6_) Polarizing microscopy results of *Candida albicans* treated with the positive control drugs (fluconazole and terbinafine) and the target compounds (**5**, **6** and **8**) at the specific concentration of 8 mg mL. (B_1–4_) TEM results for *Candida albicans* treated with the target compound (**8**) at the specific concentration of 8 mg mL^−1^.

The Royal Society of Chemistry apologises for these errors and any consequent inconvenience to authors and readers.

