# Supplementary material for: Construction of antifungal dual-target (SE, CYP51) pharmacophore models and the discovery of novel antifungal inhibitors
Source: RSC Adv. 2019 Aug 22;9(45):26302–14. doi: 10.1039/c9ra03713f (PMC9070380; doi:10.1039/c9ra03713f)
Supplement: RA-009-C9RA03713F-s001 [file RA-009-C9RA03713F-s001.pdf]

Electronic Supplementary Material (ESI) for RSC Advances.
This journal is © The Royal Society of Chemistry 2019

Supporting Information

Construction of the antifungal dual-target (SE, CYP51) pharmacophore model and the discovery of novel antifungal inhibitors

Yue Dong [a], Min Liu [a], Jian Wang [b], Ding Zhuang [a], Bin Sun [a] *

[a]*Institute of BioPharmaceutical Research, Liaocheng University, 1 Hunan Road, Liaocheng 252000, PR Chinac*

[b]*Key Laboratory of Structure-Based Drug Design & Discovery of Ministry of Education, School of Pharmaceutical Engineering,Shenyang Pharmaceutical University, 103 Wenhua Road, Shenhe District, Shenyang 110016, PR China*

*Corresponding anthor: Bin Sun, E-mail: goengoy@163.com

1H NMR, 13NMR spectra and HRMS of **5**

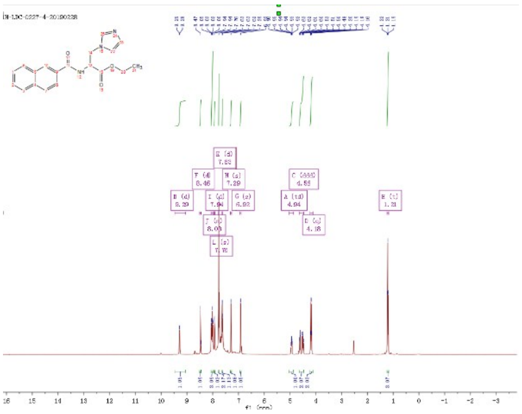

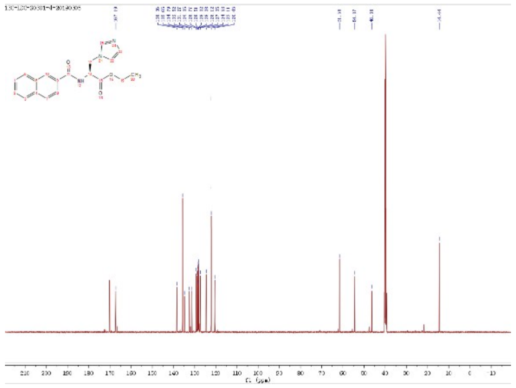

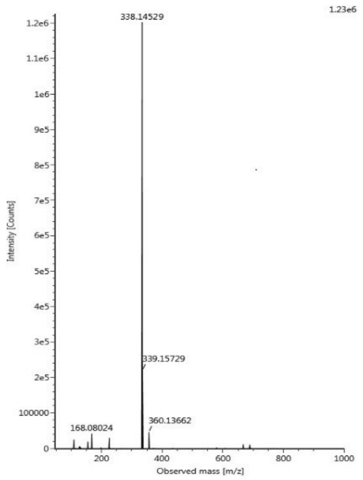

1H NMR, 13NMR spectra and HRMS of **6**

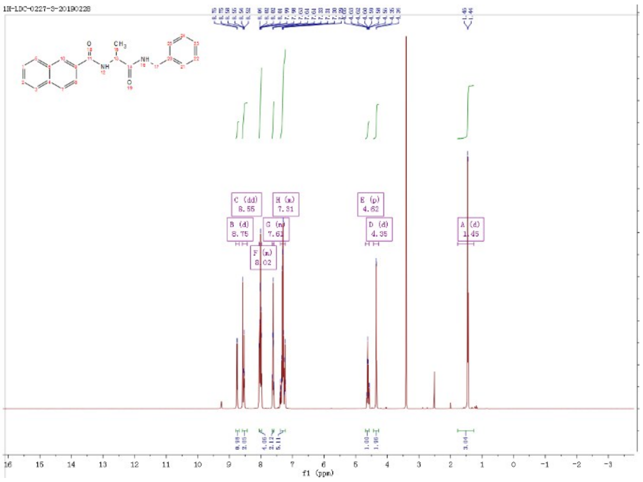

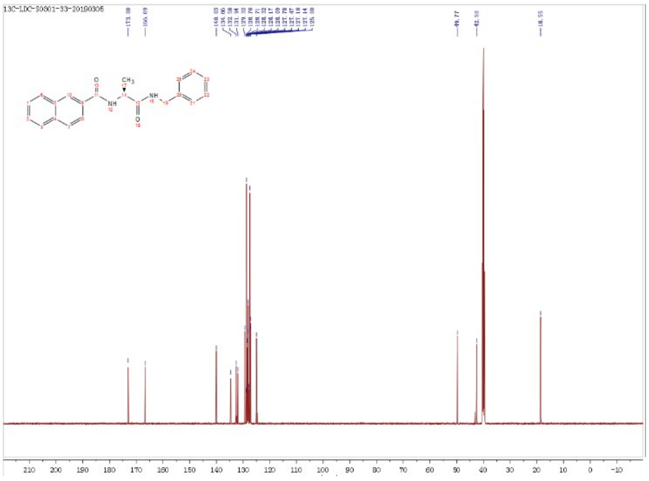

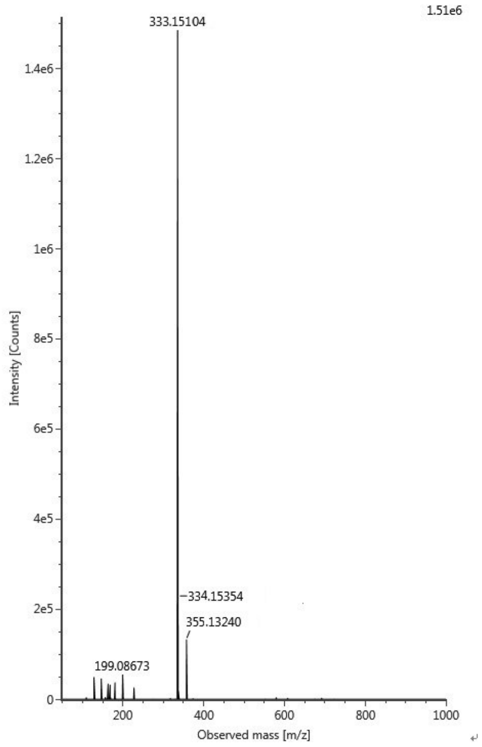

1H NMR, 13NMR spectra and HRMS of **8**

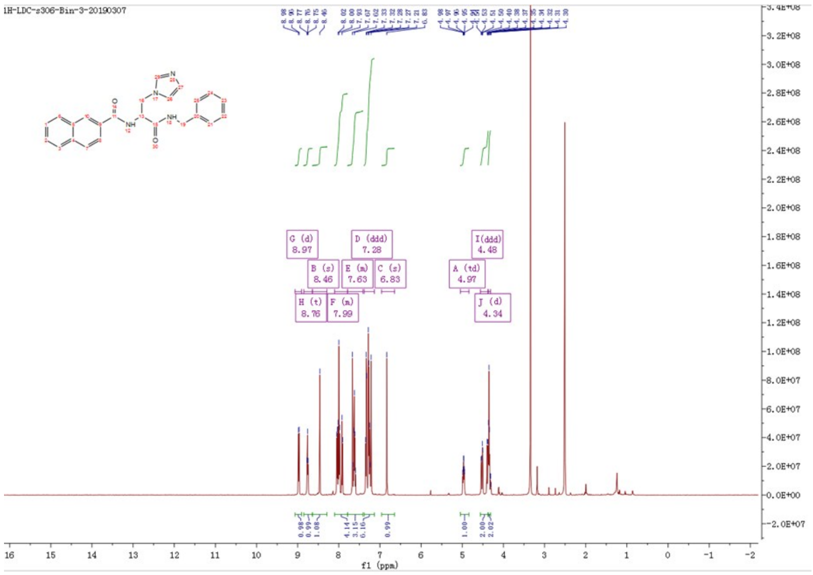

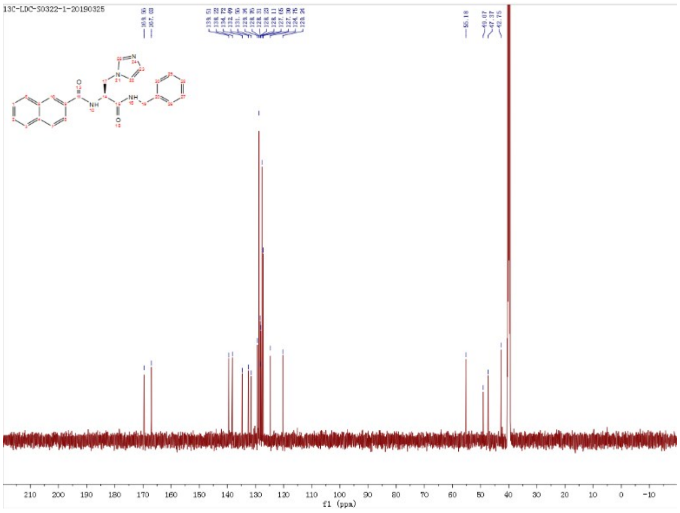
13C-LDC-S0322-1-20190325
169.56
167.03
139.51
138.22
134.72
132.49
131.56
129.36
128.76
128.31
128.23
128.11
127.65
127.30
124.75
120.26
55.18
49.07
47.37
42.75
NH
NH
O
O
N
N
210 200 190 180 170 160 150 140 130 120 110 100 90 80 70 60 50 40 30 20 10 0 -10
f1 (ppm)

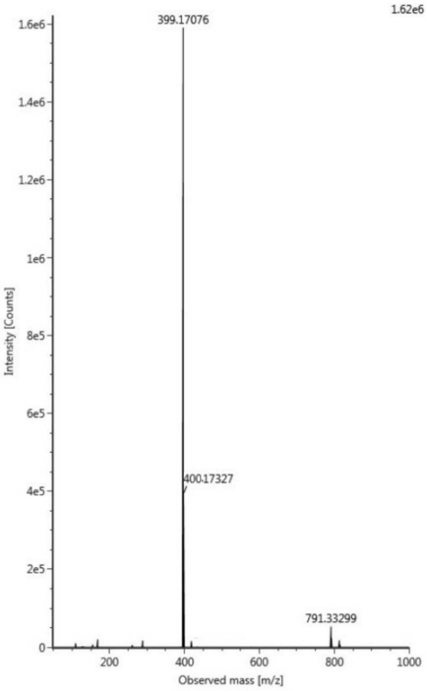
1.62e6
399.17076
400.17327
791.33299
1.6e6
1.4e6
1.2e6
1e6
8e5
6e5
4e5
2e5
0
Intensity [Counts]
200
400
600
800
1000
Observed mass [m/z]
